# Insulin Resistance Is Not Associated With Perceived Exertion or Cerebral Oxygenation During Exercise in Women

**DOI:** 10.1155/tsm2/7516755

**Published:** 2026-05-31

**Authors:** Juuso Rissanen, Antti-Pekka E. Rissanen, Timo Lakka, Maritta Pöyhönen-Alho, Aila Tiitinen, Heikki O. Tikkanen, Robert Petrella, J. Kevin Shoemaker, Juha E. Peltonen

**Affiliations:** ^1^ Institute of Clinical Medicine, Faculty of Medicine, University of Eastern Finland, Kuopio, Finland, uef.fi; ^2^ Sports and Exercise Medicine, Faculty of Medicine, University of Helsinki, Helsinki, Finland, helsinki.fi; ^3^ HULA—Helsinki Sports and Exercise Medicine Clinic, Foundation for Sports and Exercise Medicine, Helsinki, Finland; ^4^ Institute of Biomedicine, School of Medicine, University of Eastern Finland, Kuopio, Finland, uef.fi; ^5^ Department of Obstetrics and Gynecology, University of Helsinki and Helsinki University Hospital, Helsinki, Finland, helsinki.fi; ^6^ Department of Family Practice, Faculty of Medicine, The University of British Columbia, Vancouver, British Columbia, Canada, ubc.ca; ^7^ School of Kinesiology, The University of Western Ontario, London, Ontario, Canada, uwo.ca

**Keywords:** cardiopulmonary exercise test, homeostasis model assessment of insulin resistance, near-infrared spectroscopy, physical exercise, rating of perceived exertion, woman

## Abstract

Insulin resistance (IR) may jeopardise cerebral oxygenation and increase perceived exertion during exercise. We investigated whether IR is independently associated with perceived exertion or cerebral oxygenation during exercise and whether perceived exertion is associated with cerebral oxygenation during exercise. 48 premenopausal apparently healthy women underwent a cardiopulmonary exercise test (CPET) in this retrospective cross‐sectional study. We assessed IR with the homeostasis model assessment (HOMA‐IR). In addition to basic CPET data, we quantified cardiac function (impedance cardiography) and prefrontal cerebral oxygenation (near‐infrared spectroscopy [NIRS]) during CPET. We divided the subjects into women with HOMA‐IR< 3.0 (HOMA_LOW_) vs. women with HOMA‐IR≥ 3.0 (HOMA_HIGH_), and into women with lower RPE/MET (rating of perceived exertion/metabolic equivalent) slope (RPE/MET_LOW_) vs. women with higher RPE/MET slope (RPE/MET_HIGH_). RPE or NIRS responses to exercise did not differ between HOMA‐IR groups. NIRS responses to exercise did not differ between RPE/MET groups. NIRS responses did not correlate with HOMA‐IR or RPE/MET slope in all subjects. In multivariate regression analyses, a higher RPE/MET slope was predicted only by higher body mass index and weaker lung function. In conclusion, we found no associations between IR, cerebral oxygenation, and perceived exertion, whereas body mass index and lung function were independently associated with exercise‐related perceived exertion.

## 1. Introduction

Insulin resistance (IR) is a condition where the target organs’ sensitivity to respond to insulin is decreased [1]. IR together with pancreatic β‐cell dysfunction can lead to hyperglycaemia and Type 2 diabetes (T2D) [2]. Impaired glucose tolerance (IGT), characterised by IR, and T2D are major global health problems with prevalences of 374 million in 2019 (IGT) and 537 million in 2021 (T2D), as well as expected prevalences of 548 million (IGT) and 783 million (T2D) in 2045 [3, 4]. Lifestyle modifications such as physical activity are cornerstones of prevention and treatment of T2D [5]. Unfortunately, adherence to regular physical activity is poor among these groups [6, 7]. While this poor adherence is due to numerous aetiologies [8], it may also be that individuals with IR or T2D experience increased perceived exertion or exercise intolerance [9], which acts as a barrier to physical activity.

Evidence that T2D independently affects perceived exertion during exercise is controversial. Huebschmann et al. [10] reported a higher rating of perceived exertion (RPE) during submaximal cycling in adult women with T2D compared to women with no diabetes. On the contrary, another study from Huebschmann et al. [11] did not find statistically significant differences in RPE during submaximal cycling between 50‐ and 75‐year‐old sedentary women with and without T2D. Kim et al. [12] examined RPE during incremental cycling in ∼60‐year‐old males and found no significant differences at relative work rates of 25%–100% between males with and without T2D. These findings show that the limited evidence on perceived exertion and exercise intolerance in patients with T2D is controversial. Importantly, similar studies in subjects with IR not yet proceeding to T2D are yet to be conducted, and thus, the independent effect of IR on perceived exertion and exercise tolerance remains unknown. In addition, pathophysiological mechanisms behind potentially increased perceived exertion and exercise intolerance related to IR and T2D are incompletely understood [9].

Regarding mechanisms of potential exercise intolerance in IR and T2D, O_2_ uptake (V̇O_2_) kinetics at the onset of exercise is slowed in patients with T2D [9], which ‘sows the seeds for exercise intolerance’ by increasing exercise‐induced O_2_ deficit within active muscles [13]. Slowed V̇O_2_ kinetics is linked with impaired muscle O_2_ availability, which leads to increased perceived exertion as greater efferent motor drive is needed to maintain a given level of muscle force production [9].

Inadequate cerebral oxygenation may prevent efferent motor drive from increasing or even being maintained [14], and evidence from patients with interstitial lung disease, who suffer from arterial hypoxaemia, particularly during exercise, demonstrates a link between inadequate cerebral oxygenation and increased perceived exertion [15]. To understand potential limitations on cerebral oxygenation during exercise, main regulators of cerebral blood flow, which are arterial blood gases (particularly partial pressure of arterial CO_2_ [PaCO_2_]), cerebral metabolism, arterial blood pressure, neurogenic activity and cardiac output (CO) [16], need to be considered: First, regarding associations between these regulators and IR, we are not aware of reports of exercise‐related hypocapnia or hypoxaemia associated with glucose–insulin homeostasis; instead, cerebrovascular reactivity has been reported to be reduced in IR, which may lower cerebral CO_2_‐induced vasodilation [17]. Second, cerebral metabolism during exercise does not seem to be perturbed in patients with T2D [12]. Third, blood pressure response to exercise is exaggerated in individuals with IR [18], which may even increase cerebral perfusion pressure and thus cerebral oxygenation during exercise. Finally, no consistent signs of diminished CO in individuals with T2D [9] or disturbed convective O_2_ delivery in individuals with IR [19] have been observed at submaximal exercise intensities. Beyond these regulators, IR is proposed to have negative effects on cerebrovascular function by causing endothelial dysfunction and impaired insulin‐mediated vasodilation, also during exercise [20]. To summarise, in individuals with IR, potential mechanisms jeopardising adequate cerebral blood flow and cerebral oxygenation during exercise are impairments of cerebrovascular reactivity, endothelial function and insulin‐mediated vasodilation. However, it is unknown whether cerebral oxygenation is compromised during exercise among individuals with IR, or whether this is associated with perceived exertion during exercise.

The purpose of this retrospective cross‐sectional study was to examine associations of IR to perceived exertion and cerebral oxygenation during acute incremental dynamic exercise. We specifically examined: (1) Is IR associated with perceived exertion during acute incremental cycling? (2) Is IR associated with cerebral oxygenation during acute incremental cycling? (3) Is perceived exertion associated with cerebral oxygenation during acute incremental cycling?

## 2. Methods

### 2.1. Ethical Approval

Written informed consent prior to their participation in the study projects was given by all subjects. The projects conformed to the Declaration of Helsinki and were approved by the Ethics Committee of the Hospital District of Helsinki and Uusimaa, Helsinki, Finland (project‐specific identifiers: 300/E9/06, 384/13/03/00/2015, 93/13/03/03/2014).

### 2.2. Subjects

This is a retrospective observational study, in the final analyses of which a total of 144 women were assessed for inclusion. The women had participated in one of three study projects at Sports and Exercise Medicine, Faculty of Medicine, University of Helsinki, Helsinki, Finland: Autonomic Nervous System and EXErcise in gestational diabetes (ANS‐EXE) (ClinicalTrials.gov: NCT01675271), Motivation Makes the Move! (MoMaMo!) (ClinicalTrials.gov: NCT02686502) or Well‐Being of WOMen during prEgnancy: focus on individualised EXercise training (WEBWOMEX) (nonregistered). While all three projects included a lifestyle intervention, only cross‐sectional preintervention data were used for this study. The inclusion criteria of this study were aged between 18 and 40 years, body mass index (BMI) between 18.5 and 40 kg/m^2^, Caucasian ethnicity, available concentrations of fasting plasma glucose (fP‐Gluc) and fasting serum insulin (fS‐Insu), and collected data on ventilatory gas exchange, impedance cardiography and cerebral near‐infrared spectroscopy (NIRS). The study flowchart is presented in Supporting Information 1, including the exclusion criteria of the study and the overall process of subject inclusion; eventually, 48 women were included in the analyses.

### 2.3. Study Protocol

In terms of each of the three study projects, the data for the analyses of this study were collected during two different laboratory visits. Before both visits, the subjects abstained from physical exercise for at least 12 h and alcohol for at least 24 h. The first visit consisted of pre‐exercise measurements and a cardiopulmonary exercise test (CPET), which were executed 2‐3 h after a meal. Personal health and medical history were inquired about by a preliminary questionnaire; the questionnaire included a question asking to quantify leisure‐time physical activity (LTPA) as previously reported [21]. Hip and waist circumferences, and height were measured, and body mass and composition were determined by the bioimpedance method (InBody 720, Biospace Co., Ltd., Seoul, South Korea). Haemoglobin concentration ([Hb]) was analysed from a capillary blood sample from the fingertip by a blood gas analyser (ABL725, Radiometer, Copenhagen, Denmark). 12‐lead electrocardiography (ECG), blood pressure measurement and flow‐volume spirometry (Medikro Spiro 2000, Medikro Oy, Kuopio, Finland) were also included in pre‐exercise measurements at rest. Flow‐volume spirometry results were evaluated in relation to the reference values for Finnish adults [22]. Every subject’s suitability to perform CPET was ensured by a physician, after which the subjects performed CPET (see 2.4 CPET).

For the second visit to the laboratory, the subjects accomplished overnight fasting. Fasting venous blood was collected for measurements of glucose–insulin homeostasis (i.e., fP‐Gluc and fS‐Insu) and lipid profile (i.e., high‐density lipoprotein (HDL), low‐density lipoprotein (LDL), total cholesterol and triglycerides). fP‐Gluc was determined by the hexokinase method, and fS‐Insu by the immunochemiluminometric assay. The level of IR was evaluated by the homeostasis model assessment of IR (HOMA‐IR) [23]: HOMA‐IR = fP‐Gluc (mmol/L) × fS‐Insu (µU/mL)/22.5.

### 2.4. CPET

The subjects performed CPET on a cycle ergometer (Monark Ergomedic 839E, Monark Exercise AB, Vansbro, Sweden). First, subjects had a 5‐min seated rest, which was followed by a 5‐min baseline unloaded cycling. After that, a step incremental protocol (+30 W/3 min) was initiated with a work rate of 30 W, and the subjects continued cycling until voluntary exhaustion. The mean duration of the ‘exercise part’ (i.e., time from the beginning of the 5‐min unloaded cycling until the end of cycling) of the performed tests was 22 min 17 s ± 3 min 15 s, with a range from 16 min 9 s to 33 min. We measured breath‐by‐breath ventilation and alveolar gas exchange, asked RPE, monitored heart rate (HR) and the electrical activity of the heart, evaluated left ventricular stroke volume (SV) and then calculated several other respiratory‐ and cardiovascular‐related variables based on the collected data; please see Supporting Information 2 for the detailed description of the applied methods.

### 2.5. Cerebral Oxygenation

Cerebral oxygenation was measured by a spatially resolved continuous wave NIRS device (Oxymon Mk III Near‐Infrared Spectrophotometer, Artinis Medical Systems, Zetten, The Netherlands). Three transmitting and one receiving NIRS optodes were placed at the level of the lateral prefrontal cortex (LPFC), approximately 2 cm above the right eyebrow. This site of the prefrontal cortex has been linked with planning [24] and pacing [25] of voluntary movements and may also make a contribution to exercise (in)tolerance and termination [26]. The optodes were housed in an optically dense plastic holder attached to the skin using double‐sided adhesive tape and a special headband. The inter‐optode distances of 35 to 50 mm were chosen to ensure that a good signal quality was reached before starting the measurements. The optodes operated at wavelengths of 765 and 860 nm, and the data were collected at a sampling rate of 10 Hz.

The theory of NIRS and its applications in exercise physiology have been described elsewhere [27]. Briefly, the intensity of incident and transmitted light was recorded continuously and, along with the specific optical pathlength and extinction coefficients, used for online estimation and display of relative concentration changes of deoxygenated Hb (ΔHHb), oxygenated Hb (ΔO_2_Hb) and total Hb (Δ[tHb] = Δ[HHb] + Δ[O_2_Hb]). Differential pathlength factor (DPF) was calculated according to the manufacturer’s guidelines (DPF = 4.99 + 0.067 × Age^0.814^). The light attenuation slope, consisting of the distance from the three emitting points as detected by the sensor of the receiving optode, was used to calculate tissue saturation index (TSI = Δ[O_2_Hb]/Δ[tHb] × 100%). The procured NIRS data were averaged to give values in 1‐s intervals and time‐aligned with cardiopulmonary data.

The NIRS data were also presented as normalised relative concentration changes of HHb and tHb (i.e., % Δ[HHb] and %Δ[tHb], respectively): The normalised values were formulated so that 0% represents the minimum average of the last 30 s of any work rate, 100% represents the maximum average of the last 30 s of any work rate, and percentage values for each work rate represent values in relation to the 0%–100% spectrum.

### 2.6. Statistical Analyses

The subjects were divided into two groups in two ways. First, to assess whether IR was associated with the outcomes of interest (e.g., RPE and cerebral oxygenation), the subjects were divided into two groups as follows: a HOMA_LOW_ group with HOMA‐IR < 3.0 and a HOMA_HIGH_ group with HOMA‐IR ≥ 3.0. The threshold of 3.0 was chosen as it identifies the highest quartile within individuals without existing diabetes [28]. Second, to assess whether the exercise‐induced increase of RPE in relation to metabolic equivalents (MET; 1 MET = 3.5 mL·min^−1^·kg^−1^ of V̇O_2_) was associated with the outcomes of interest (e.g., HOMA‐IR and cerebral oxygenation), the subjects were divided into two groups as follows: Individual linear regression lines of RPE over MET were first drawn for each subject based on their CPET data, and the slopes of each individual’s regression lines were determined. Each RPE/MET regression line was drawn from unloaded cycling to the individual’s last accomplished work rate; peak exercise data were not included, because the inclusion of the peak values would have tended to either plateau or steepen the lines near peak exercise and thus distort the linearity of the slopes. The median of these RPE/MET slopes was then determined and equalled 1.91, and the subjects were eventually divided into a RPE/MET_LOW_ group with RPE/MET slope < 1.91 and a RPE/MET_HIGH_ group with RPE/MET slope ≥ 1.91. Furthermore, we performed sensitivity analyses to assess if there were any differences in cerebral (de)oxygenation responses during exercise between the subjects belonging to the low and high extremities of HOMA‐IR or RPE/MET slope spectra: As such sensitivity analyses we compared (1) the group with HOMA‐IR < 2.0 (*n* = 24) to the group with high HOMA‐IR > 3.0 (*n* = 13) and (2) the subjects belonging to the lowest tertile of RPE/MET slope (*n* = 16, RPE/MET slope < 1.66) to the subjects belonging to the highest tertile of RPE/MET slope (*n* = 16, RPE/MET slope > 2.30).

Data are expressed as mean ± standard deviation (SD) or mean (95% confidence interval [CI]) for normally distributed data and median (interquartile range [IQR]) for non‐normally distributed data. Work rate‐specific values for each variable of interest were determined as the averages of the last 30 s of each work rate, including seated rest and peak exercise, except for peak V̇O_2_ (V̇O_2peak_), which was determined as the highest 60‐s moving average interval.

Normality was tested by using the Shapiro–Wilk test, and data were ln‐transformed when needed. One‐way ANOVA was used to compare descriptive characteristics and peak exercise values between groups (HOMA_LOW_ vs. HOMA_HIGH_ and RPE/MET_LOW_ vs. RPE/MET_HIGH_). Two‐way repeated‐measures ANOVA was used to evaluate potential differences in CPET responses between HOMA‐IR and RPE/MET groups: Group × Exercise interactions were evaluated, while Group (i.e., HOMA_LOW_ vs. HOMA_HIGH_, or RPE/MET_LOW_ vs. RPE/MET_HIGH_) was a between‐subjects factor, and Exercise (i.e., rest, unloaded cycling, work rates accomplished by each subject [30, 60, 90 W], peak exercise) was a within‐subject factor. In a separate analysis, we used relative work rates (i.e., % of peak work rate; rest [0%], 25%, 50%, 75% and 100% of peak exercise) as a within‐subject factor. A post hoc Bonferroni analysis was performed, if a significant interaction was observed. Pearson’s correlation coefficients were evaluated to determine whether BMI was significantly associated with cerebral oxygenation parameters and whether it was thus necessary to use BMI as a covariate when comparing cerebral oxygenation parameters between RPE/MET groups. The Mann–Whitney *U* test was used for non‐normally distributed data.

Furthermore, univariate linear regression analyses were used to assess whether cerebral oxygenation parameters at the relative work rate of 50% were associated with HOMA‐IR. Univariate linear regression analyses were also used to examine associations between RPE/MET slope and its potential predictors, including age, LTPA, BMI, HOMA‐IR, forced expired volume in 1 s (FEV1, as it differed significantly between RPE/MET groups) and cerebral oxygenation parameters at the relative work rate of 50%. A multivariate backward stepwise linear regression analysis was then performed to identify the predictors that were independently associated with RPE/MET slope; candidate predictors for entering the first step of the backward stepwise analysis were the predictors showing a statistically significant (*p* < 0.05) univariate association with RPE/MET slope.

Statistical significance was set at *p* < 0.05. The results were computed with IBM SPSS Statistics 27 (IBM Corporation, Armonk, USA).

## 3. Results

### 3.1. Descriptive Characteristics

Table 1 presents the descriptive characteristics of all subjects and each group. HOMA_HIGH_ had higher body mass, BMI, body fat, waist circumference, fP‐Gluc, fS‐Insu, HOMA‐IR, triglycerides and diastolic arterial pressure at seated rest, and lower HDL cholesterol than HOMA_LOW_. The two HOMA‐IR groups did not differ in terms of other descriptive characteristics.

**TABLE 1:** Descriptive characteristics.

	**ALL (n = 48)**	**HOMA_LOW_ (n = 34)**	**HOMA_HIGH_ (n = 14)**	**p**	**RPE/MET_LOW_ (n = 24)**	**RPE/MET_HIGH_ (n = 24)**	**p**

Age (years)	31.5 ± 4.8	32.1 ± 4.6	29.8 ± 5.2	0.122	32.9 ± 4.7	30.0 ± 4.6	0.031
LTPA (h:min·wk^−1^)	2:42 ± 1:23^a^	2:42 ± 1:24^b^	2:42 ± 1:25^c^	0.988	2:51 ± 1:30^d^	2:33 ± 1:17	0.458
Body size and composition							
Height (cm)	167 ± 8	167 ± 8	168 ± 8	0.561	166 ± 8	168 ± 8	0.557
Body mass (kg)	83 ± 19	78 ± 18	95 ± 13	0.001	75 ± 18	91 ± 16	0.002
BMI (kg·m^−2^)	29.5 ± 5.5	27.9 ± 5.4	33.5 ± 3.1	< 0.001	26.9 ± 5.8	32.1 ± 3.7	0.001
Body fat (%)	38 ± 9	35 ± 9	44 ± 5	0.002	33 ± 9	43 ± 6	< 0.001
Waist (cm)	93 ± 13^e^	89 (79–101)^b^	103 (96–106)	0.009	88 ± 14^f^	98 ± 9	0.005
Spirometry							
FVC (L)	4.0 ± 0.6	4.1 ± 0.6	4.0 ± 0.6	0.657	4.2 ± 0.7	3.9 ± 0.5	0.240
FVC (z‐score)	−0.17 ± 1.13	−0.07 ± 1.15	−0.44 ± 1.09	0.318	0.14 ± 1.21	−0.50 ± 0.98	0.050
FEV1 (L)	3.3 ± 0.5	3.3 ± 0.5	3.3 ± 0.5	0.680	3.4 ± 0.4	3.2 ± 0.5	0.257
FEV1 (z‐score)	−0.36 ± 0.95	−0.34 ± 0.98	−0.40 ± 0.91	0.828	−0.02 ± 0.90	−0.69 ± 0.89	0.014
FEV1/FVC ratio (%)	81 ± 7	81 ± 8	84 ± 4	0.174	80 (78–85)	83 (78–86)	0.409
FEV1/FVC ratio (z‐score)	−0.31 ± 1.39	−0.45 ± 1.54	0.03 ± 0.90	0.284	−0.26 (−1.01–0.49)	−0.13 (−1.06–0.70)	0.695
Blood samples							
[Hb] (g·L^−1^)	135 ± 7	134 ± 7	137 ± 7	0.219	134 ± 6	136 ± 8	0.516
Fasting glucose (mmol·L^−1^)	5.2 ± 0.5	5.1 ± 0.4	5.6 ± 0.4	< 0.001	5.2 ± 0.6	5.3 ± 0.3	0.543
Fasting insulin (µU·mL^−1^)	10.1 ± 6.0	6.5 (4.2–10.4)	16.9 (13.2–20.3)	< 0.001	7.8 ± 4.6	12.4 ± 6.4	0.007
HOMA‐IR	2.4 ± 1.5	1.4 (1.0–2.4)^j^	4.2 (3.3–5.1)^j^	< 0.001	1.9 ± 1.3	2.9 ± 1.6	0.015
HDL cholesterol (mmol·L^−1^)	1.5 ± 0.4^g^	1.6 ± 0.4^h^	1.3 ± 0.3	0.011	1.6 ± 0.4^i^	1.4 ± 0.4	0.066
LDL cholesterol (mmol·L^−1^)	2.9 ± 0.8^g^	2.9 ± 0.8^h^	2.9 ± 0.7	0.948	3.0 ± 0.9^i^	2.9 ± 0.6	0.714
Total cholesterol (mmol·L^−1^)	4.6 ± 0.9^g^	4.6 ± 0.9^h^	4.6 ± 0.7	0.939	4.8 ± 1.0^i^	4.5 ± 0.7	0.266
Triglycerides (mmol·L^−1^)	1.0 (0.7–1.3)^g^	0.8 (0.7–1.2)^h^ ^,^ ^j^	1.3 (1.0–1.7)^j^	0.002	0.9 (0.7–1.2)^i^ ^,^ ^j^	1.0 (0.7–1.6)^j^	0.278
Blood pressure at seated rest							
SAP (mmHg)	117 ± 14^e^	116 ± 15^b^	118 ± 13	0.715	117 ± 15^f^	117 ± 13	0.966
DAP (mmHg)	81 ± 11^e^	78 ± 10^b^	88 ± 10	0.003	81 ± 10^f^	81 ± 11	0.978

*Note:* Data are means ± SD for normally distributed variables or median (IQR) for non‐normally distributed variables.

Abbreviations: [Hb], haemoglobin concentration; BMI, body mass index; DAP, diastolic arterial pressure; FEV1, forced expired volume in 1 s; FVC, forced vital capacity; HOMA‐IR, homeostasis model assessment index of insulin resistance; IQR, interquartile range; LTPA, leisure‐time physical activity; SAP, systolic arterial pressure; SD, standard deviation.

^a^ = 46.

^b^ = 33.

^c^ = 13.

^d^ = 22.

^e^ = 47.

^f^ = 23.

^g^ = 44.

^h^ = 30.

^i^ = 20.

^j^ = Ln transformed for statistical analysis due to non‐normally distributed data.

The RPE/MET_HIGH_ had higher body mass, BMI, body fat, waist circumference, fS‐Insu and HOMA‐IR than the RPE/MET_LOW_. RPE/MET_HIGH_ group was younger than RPE/MET_LOW_. In addition, RPE/MET_HIGH_ had lower FEV1 (z‐score) than RPE/MET_LOW_. No other differences were observed between the two RPE/MET groups.

### 3.2. CPET

Peak work rates and respiratory and cardiovascular responses at peak exercise of all subjects and each group are detailed in Table 2. The responses of key variables, which regulate cerebral blood flow during exercise [16], to CPET in HOMA‐IR groups are presented in Figure 1. The responses of RPE to CPET in HOMA‐IR groups are presented in Figure 2.

**TABLE 2:** Work rates and respiratory and cardiovascular responses at peak exercise.

	**HOMA**_**LOW**_ **(*n* = 34)**	**HOMA**_**HIGH**_ **(*n* = 14)**	**p**	**RPE/MET**_**LOW**_ **(*n* = 24)**	**RPE/MET**_**HIGH**_ **(*n* = 24)**	**p**

Work rate (W)	162 (95% CI 150–174)	165 (95% CI 150–181)	0.780	169 (95% CI 154–185)	157 (95% CI 146–168)	0.196
Work rate (W·kg^−1^·FFM)	3.3 (95% CI 3.1–3.5)	3.1 (95% CI 2.9–3.4)	0.278	3.5 (95% CI 3.3–3.7)	3.0 (95% CI 2.9–3.2)	0.002
V̇O_2_ (L·min^−1^)	2.11 (95% CI 1.97–2.25)	2.18 (95% CI 1.99–2.37)	0.611	2.19 (95% CI 1.98–2.39)	2.08 (95% CI 1.96–2.19)	0.343
V̇O_2_ (mL·min^−1^·kg^−1^)	28 (95% CI 26–30)	23 (95% CI 21–25)	0.007	30 (95% CI 28–32)	23 (95% CI 22–25)	< 0.001
V̇O_2_ (mL·min^−1^·kg^−1^·FFM)	43 (95% CI 41–45)	42 (95% CI 38–45)	0.436	45 (95% CI 42–47)	41 (95% CI 38–43)	0.013
Ventilation (l·min^−1^)	88 (95% CI 81–94)	90 (95% CI 84–96)	0.604	90 (95% CI 82–98)	87 (95% CI 82–93)	0.604
Fb (breaths·min^−1^)	47 (95% CI 44–51)	44 (95% CI 40–47)	0.119	48 (95% CI 44–51)	45 (95% CI 42–48)	0.275
BR (%)	33 (95% CI 29–37)	32 (95% CI 26–37)	0.764	33 (95% CI 29–38)	31 (95% CI 27–36)	0.510
Estimated PaCO_2_ (mmHg)	36 (IQR 32–38)	36 (IQR 30–38)	0.618	35 (95% CI 33–37)	35 (95% CI 34–37)	0.943
PETCO_2_ (mmHg)	33 (95% CI 31–35)	33 (95% CI 31–35)	0.986	33 (95% CI 31–36)	33 (95% CI 32–35)	0.894
SpO_2_ (%)	97 (95% CI 96–97)	97 (95% CI 96–98)	0.968	97 (95% CI 96–97)	97 (95% CI 96–97)	0.884
CaO_2_ (mL O_2_·100 mL^−1^ blood)	17.4 (95% CI 17.0–17.7)	17.7 (95% CI 17.1–18.3)	0.262	17.4 (95% CI 17.0–17.7)	17.5 (95% CI 17.0–18.0)	0.585
HR (bpm)	182 (IQR 177–186)	182 (IQR 173–188)	0.856	181 (IQR 177–185)	184 (IQR 176–189)	0.342
HR (%)	96 (95% CI 94–98)	94 (95% CI 91–98)	0.280	96 (95% CI 94–98)	95 (95% CI 93–97)	0.433
SV (mL)	90 (95% CI 84–96)^a^	88 (95% CI 78–98)^b^	0.715	88 (95% CI 80–97)^c^	90 (95% CI 84–96)^d^	0.685
SV_allom_ (mL·kg^−0.55^·FFM)	10.7 (95% CI 10.1–11.2)^a^	10.0 (95% CI 8.94–11.1)^b^	0.235	10.7 (95% CI 9.88–11.5)^c^	10.4 (95% CI 9.73–11.0)^d^	0.505
CO (L·min^−1^)	16.2 (95% CI 15.2–17.2)^a^	15.8 (95% CI 14.1–17.4)^b^	0.640	15.8 (95% CI 14.3–17.4)^c^	16.2 (95% CI 15.3–17.2)^d^	0.643
CO_allom_ (mL·min^−1^·kg^−0.50^·FFM)	2330 (95% CI 2210–2450)^a^	2180 (95% CI 1970–2400)^b^	0.217	2320 (95% CI 2140–2510)^c^	2270 (95% CI 2140–2400)^d^	0.608
SVC (mL·min^−1^·mmHg^−1^)	143 (IQR 127–161)^a^ ^,^ ^e^	132 (IQR 124–152)^b^ ^,^ ^e^	0.423	143 (IQR 125–179)^c^ ^,^ ^e^	140 (IQR 126–159)^d^ ^,^ ^e^	0.333
SAP (mmHg)	168 (95% CI 160–175)^f^	177 (95% CI 164–190)	0.157	164 (95% CI 154–174)^g^	176 (95% CI 169–184)	0.051
DAP (mmHg)	84 (95% CI 81–88)^f^	82 (95% CI 78–86)	0.419	84 (95% CI 79–88)^g^	84 (95% CI 80–87)	0.957
MAP (mmHg)	112 (95% CI 108–116)^f^	114 (95% CI 108–119)	0.643	111 (95% CI 105–116)^g^	115 (95% CI 110–119)	0.205
C(a‐v)O_2_ (mL O_2_·100 mL^−1^ blood)	12.7 (95% CI 12.0–13.3)^a^	13.2 (95% CI 12.0–14.4)^b^	0.398	12.6 (95% CI 11.6–13.5)^c^	13.0 (95% CI 12.3–13.7)^d^	0.414
RPE	19 (IQR 18–20)	20 (IQR 19–20)	0.164	19 (IQR 18–20)	20 (IQR 19–20)	0.277
RER	1.15 (95% CI 1.13–1.17)	1.11 (95% CI 1.09–1.14)	0.067	1.15 (95% CI 1.13–1.17)	1.13 (95% CI 1.10–1.15)	0.183

*Note:* Data are mean (95% CI) for normally distributed variables and median (IQR) for non‐normally distributed variables.

Abbreviations: BR, breathing reserve; C(a‐v)O_2_, systemic arteriovenous O_2_ difference; CaO_2_, arterial O_2_ content; CI, confidence interval; CO, cardiac output; CO_allom_, cardiac output scaled allometrically to FFM; DAP, diastolic arterial pressure; Fb, frequency of breathing; FFM, fat‐free mass; HR (%), heart rate, % of age‐predicted maximal HR (=220‐age); HR (bpm), heart rate, beats per minute; IQR, interquartile range; MAP, mean arterial pressure; PaCO_2_, partial pressure of arterial carbon dioxide; PETCO_2_, end‐tidal pressure of carbon dioxide; RER, respiratory exchange ratio; RPE, rating of perceived exertion; SAP, systolic arterial pressure; SpO_2_, arterial O_2_ saturation; SV, stroke volume; SV_allom_, stroke volume scaled allometrically to FFM; SVC, systemic vascular conductance; SVCi, systemic vascular conductance index; VO_2_, pulmonary O_2_ uptake.

^a^ = 29.

^b^ = 10.

^c^ = 16.

^d^ = 23.

^e^ = Ln transformed for statistical analysis due to non‐normally distributed data.

^f^ = 33.

^g^ = 23.

FIGURE 1Pulmonary O_2_ uptake (V̇O_2_) scaled relative to fat‐free mass (FFM) (a), estimated pressure of arterial CO_2_ (PaCO_2_) (b), end‐tidal pressure of CO_2_ (PETCO_2_) (c), arterial O_2_ content (CaO_2_) (d), mean arterial pressure (MAP) (e) and cardiac output scaled allometrically to FFM (CO_allom_), as a function of work rate (W). Black circles (●) = HOMA_LOW_ (*n* = 34), white circles (○) = HOMA_HIGH_ (*n* = 14). The *p* values refer to two‐way repeated‐measures ANOVA: Group (HOMA_LOW_ vs. HOMA_HIGH_) is a between‐subjects factor, and Exercise (rest, unloaded cycling, work rates accomplished by each subject [30, 60, 90 W] and peak exercise) is a within‐subject factor. *p* ranges describe the ranges of the between‐group *p* values from minimum to maximum in the Mann–Whitney *U* test. ^∗^ *p* < 0.05. ^a^Log‐transformed data were used in a two‐way repeated‐measures ANOVA due to non‐normally distributed data. ^b^ *n* = 47. ^c^ *n* = 39.

**Figure:**
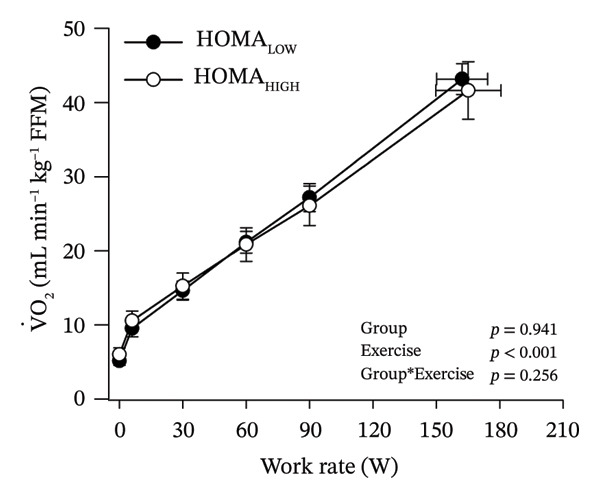
(a)

**Figure:**
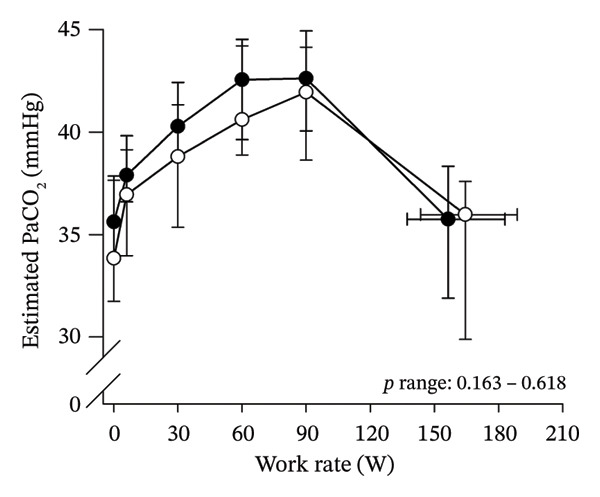
(b)

**Figure:**
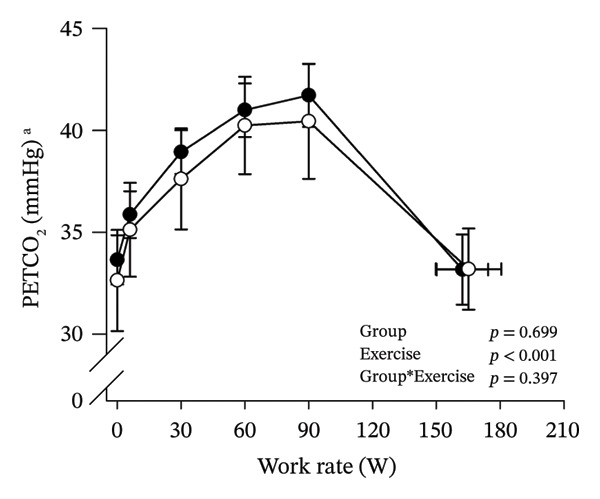
(c)

**Figure:**
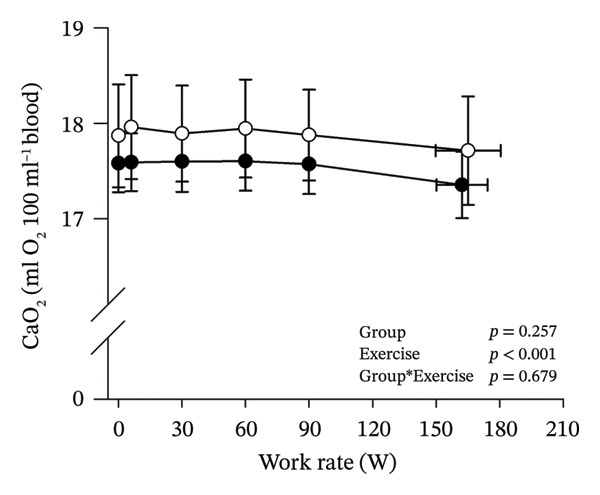
(d)

**Figure:**
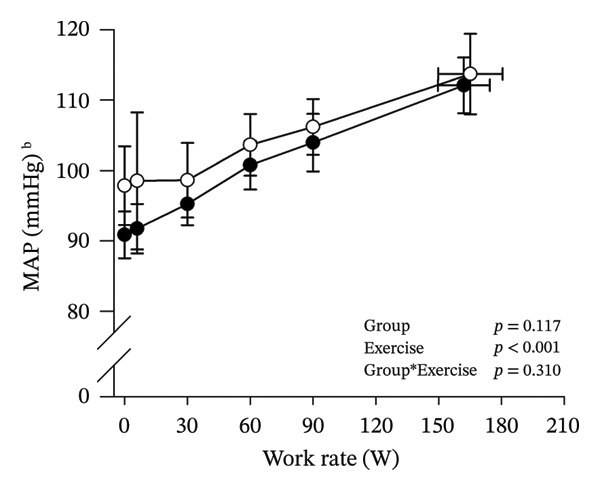
(e)

**Figure:**
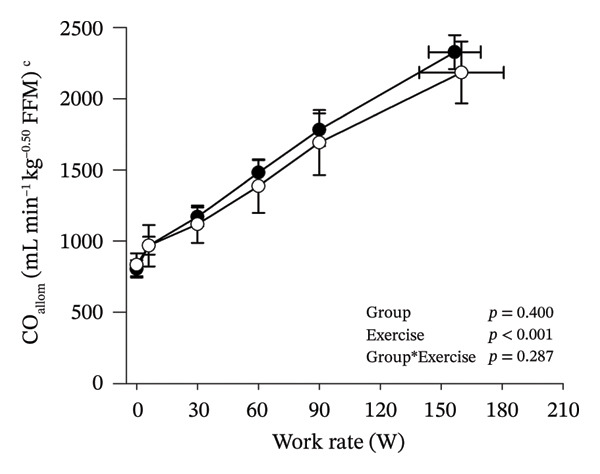
(f)

FIGURE 2Rating of perceived exertion (RPE) as a function of work rate (W) (a), RPE as a function of relative work rate (%) (b). Black circles (●) = HOMA_LOW_ (*n* = 34), white circles (○) = HOMA_HIGH_ (*n* = 14). *p* range describes the range of the *p* values from minimum to maximum in the Mann–Whitney *U* test.

**Figure:**
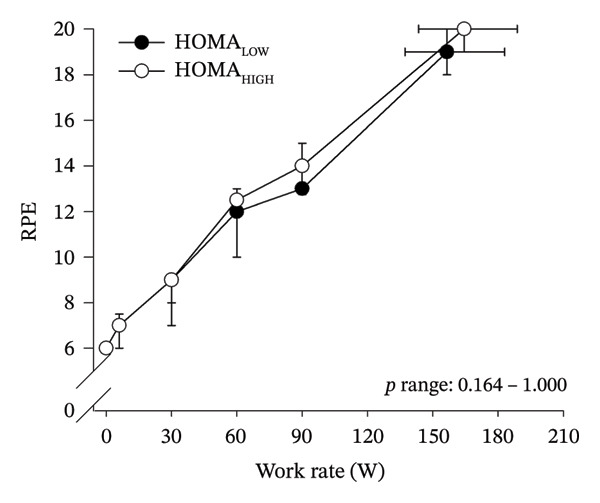
(a)

**Figure:**
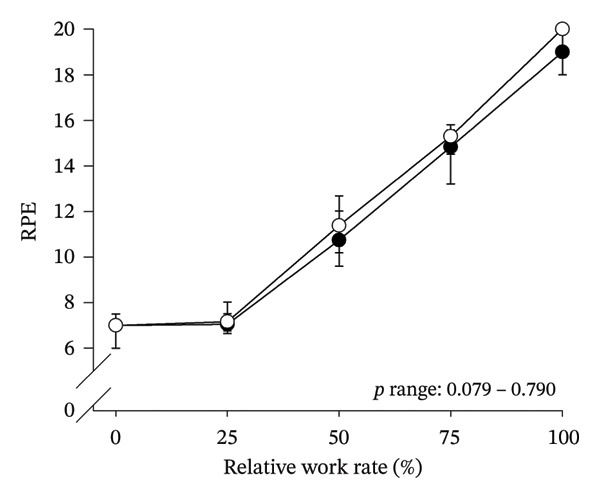
(b)

HOMA_HIGH_ had lower V̇O_2_ scaled to body mass at peak exercise than HOMA_LOW_ (Table 2). There were no differences in V̇O_2_ scaled to FFM, estimated PaCO_2_, CaO_2_, MAP, RPE or any other variables between HOMA‐IR groups; also, no significant Group × Exercise interactions were observed (Figures 1 and 2). V̇_E_/V̇CO_2_ slope did not differ between HOMA‐IR groups (HOMA_LOW_ 27.1 ± 3.5 vs. HOMA_HIGH_ 28.5 ± 3.5, *p* = 0.236). RPE/MET slope was steeper in HOMA_HIGH_ than in HOMA_LOW_ group (2.4 [IQR 1.8–2.9] vs. 1.8 [IQR 1.6–2.3], *p* = 0.004).

At peak exercise, RPE/MET_HIGH_ had lower work rate scaled to FFM, V̇O_2_ scaled to body mass and V̇O_2_ scaled to FFM compared to RPE/MET_LOW_. No other differences in peak exercise responses, such as differences in estimated PaCO_2_, CaO_2_, MAP or RPE, between RPE/MET groups were observed (Table 2).

### 3.3. Cerebral Oxygenation

Figures 3 and 4 present cerebral (de)oxygenation responses as a function of absolute and relative work rates in HOMA‐IR and RPE/MET groups, respectively. No relevant differences were observed in cerebral oxygenation during CPET between HOMA‐IR groups; HOMA_LOW_ had statistically higher Δ[HHb] at rest, but the difference was minimal as an absolute value (Figure 3(e)). RPE/MET_LOW_ had higher %Δ[tHb] than RPE/MET_HIGH_ at a work rate of 60 W (Figure 4(h)), but no other differences in cerebral oxygenation responses to CPET were observed between RPE/MET groups. It is noteworthy from a statistical perspective that although BMI differed between both HOMA‐IR groups and RPE/MET groups, we did not use BMI as a covariate when performing two‐way repeated‐measures ANOVA because it had no statistically significant associations with cerebral (de)oxygenation data (*p* > 0.05).

FIGURE 3Cerebral tissue saturation index (TSI) as a function of work rate (W) (a), relative concentration change of cerebral deoxygenated Hb (Δ[HHb]) as a function of work rate (W) (b), relative concentration change of cerebral total Hb (Δ[tHb]) as a function of work rate (W) (c), cerebral TSI as a function of relative work rate (%) (d), cerebral Δ[HHb] as a function of relative work rate (%) (e), cerebral Δ[tHb] as a function of relative work rate (%) (f), normalised relative concentration change of cerebral deoxygenated Hb (%Δ[HHb]) as a function of work rate (W) (g), normalised relative concentration change of cerebral total Hb (%Δ[tHb]) as a function of work rate (W) (h). Black circles (●) = HOMA_LOW_ (*n* = 34), white circles (○) = HOMA_HIGH_ (*n* = 14). The *p* values refer to a two‐way repeated‐measures ANOVA: Group (HOMA_LOW_ vs. HOMA_HIGH_) is a between‐subjects factor, and Exercise (panels a–b: rest, unloaded cycling, work rates accomplished by each subject [30 W, 60 W, 90 W] and peak exercise; panel d: rest and 25%–100% of peak work rate) is a within‐subject factor. *p* range describes the range of the *p* values from minimum to maximum in the Mann–Whitney *U* test.

**Figure:**
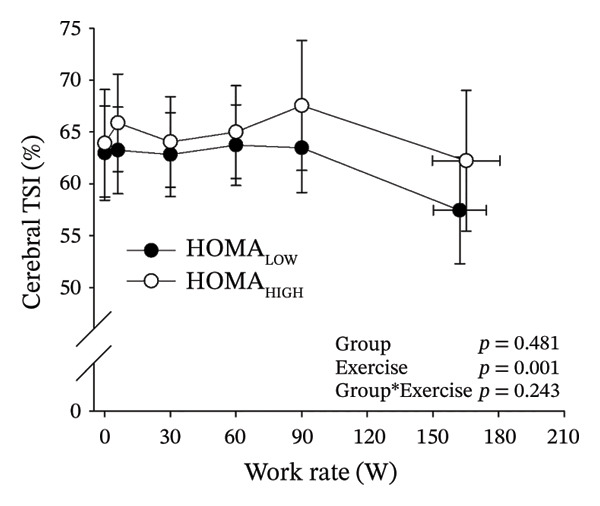
(a)

**Figure:**
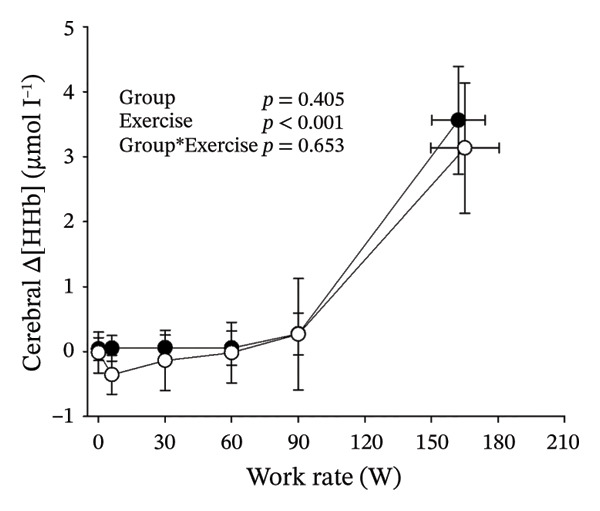
(b)

**Figure:**
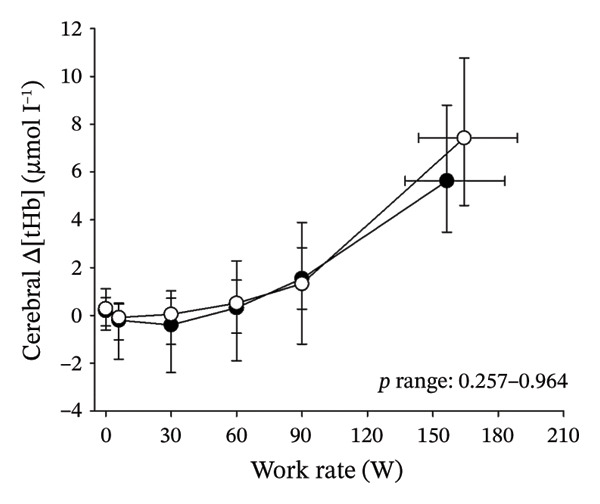
(c)

**Figure:**
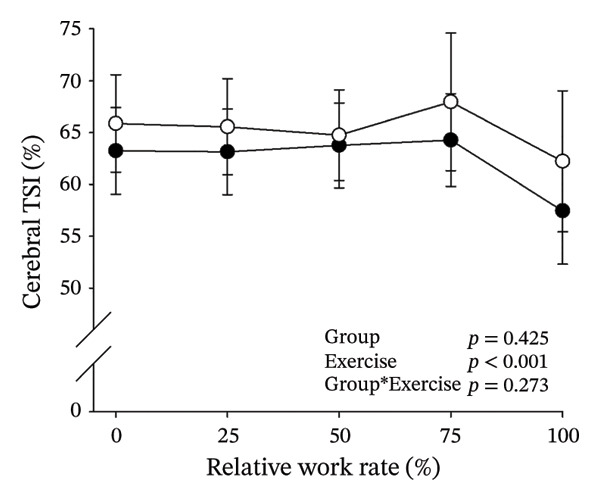
(d)

**Figure:**
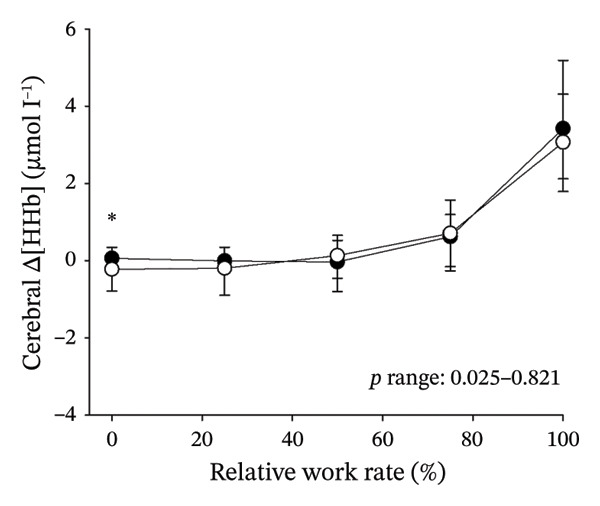
(e)

**Figure:**
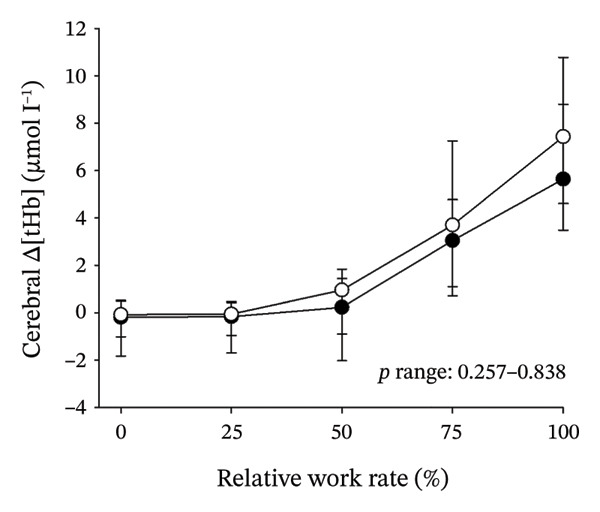
(f)

**Figure:**
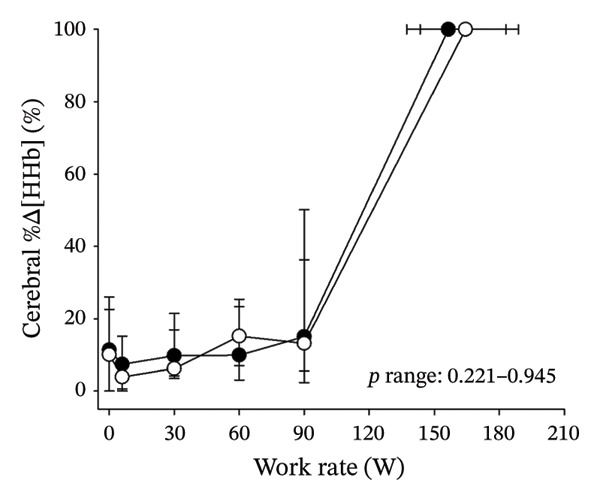
(g)

**Figure:**
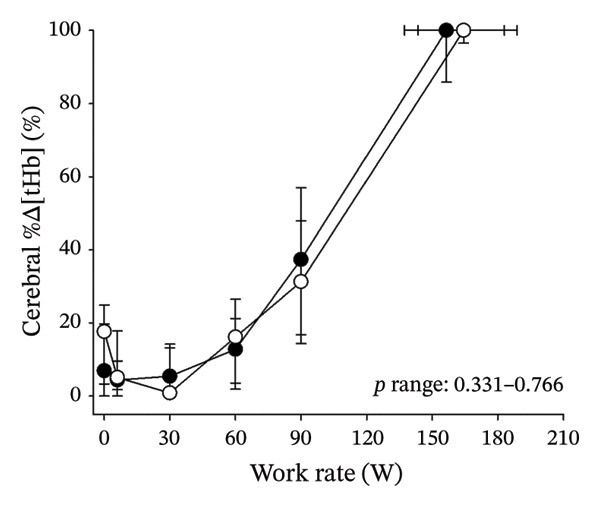
(h)

FIGURE 4Cerebral tissue saturation index (TSI) as a function of work rate (W) (a), relative concentration change of cerebral deoxygenated Hb (Δ[HHb]) as a function of work rate (W) (b), relative concentration change of cerebral total Hb (Δ[tHb]) as a function of work rate (W) (c), cerebral TSI as a function of relative work rate (%) (d), cerebral Δ[HHb] as a function of relative work rate (%) (e), cerebral Δ[tHb] as a function of relative work rate (%) (f), normalised relative concentration change of cerebral deoxygenated Hb (%Δ[HHb]) as a function of work rate (W) (g), normalised relative concentration change of cerebral total Hb (%Δ[tHb]) as a function of work rate (W) (h). Black circles (●) = RPE/MET_LOW_ (*n* = 24), white circles (○) = RPE/MET_HIGH_ (*n* = 24). The *p* values refer to a two‐way repeated‐measures ANOVA: Group (RPE/MET_LOW_ vs. RPE/MET_HIGH_) is a between‐subjects factor, and Exercise (panel a: rest, unloaded cycling, work rates accomplished by each subject [30, 60, 90] and peak exercise; panel d: rest and 25%–100% of peak work rate) is a within‐subject factor. *p* range describes the range of the *p* values from minimum to maximum in the Mann–Whitney *U* test. ^∗^ *p* < 0.05.

**Figure:**
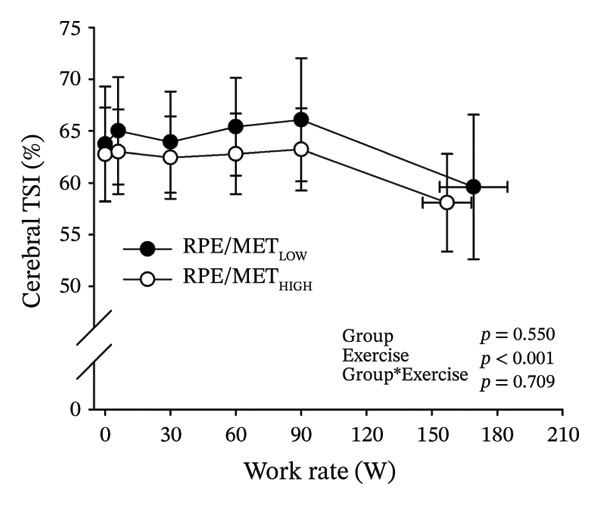
(a)

**Figure:**
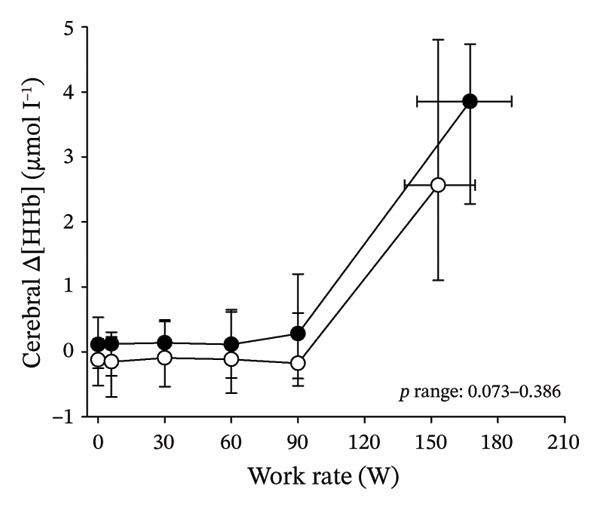
(b)

**Figure:**
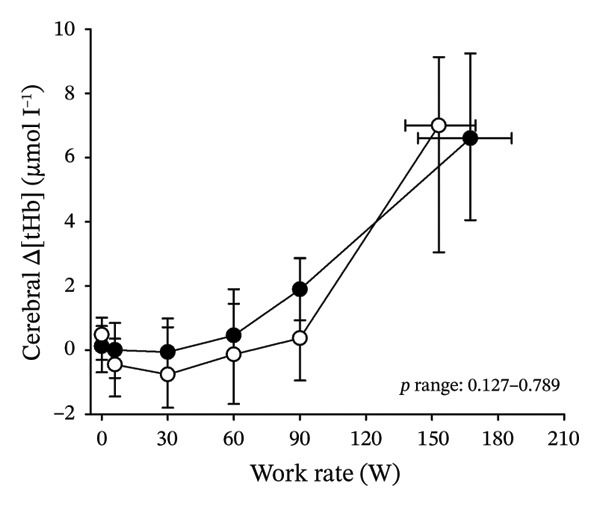
(c)

**Figure:**
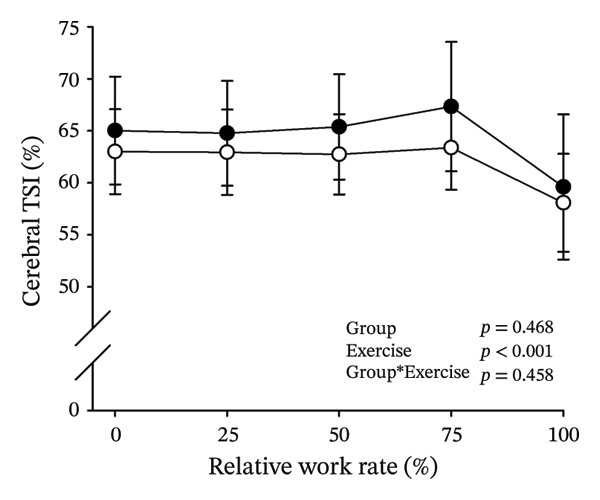
(d)

**Figure:**
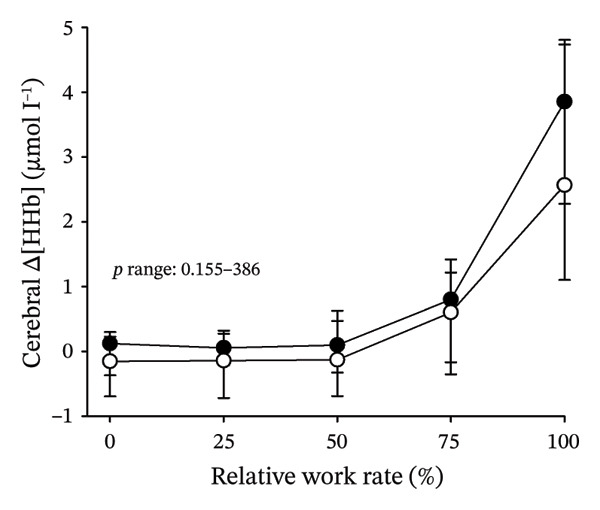
(e)

**Figure:**
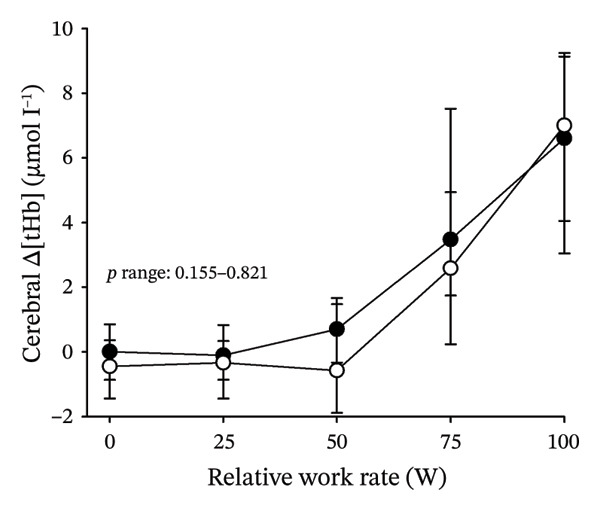
(f)

**Figure:**
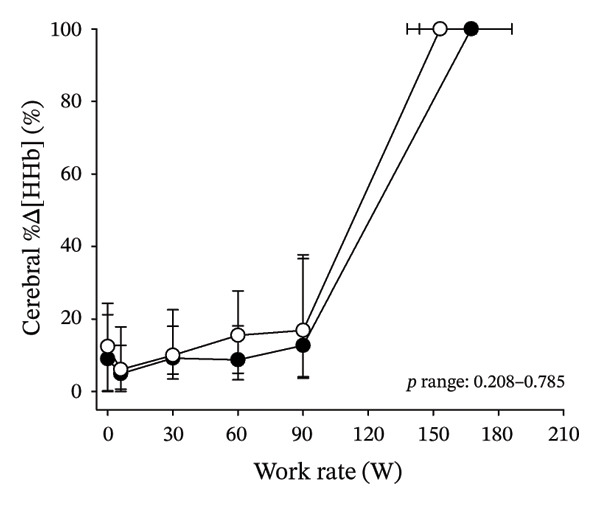
(g)

**Figure:**
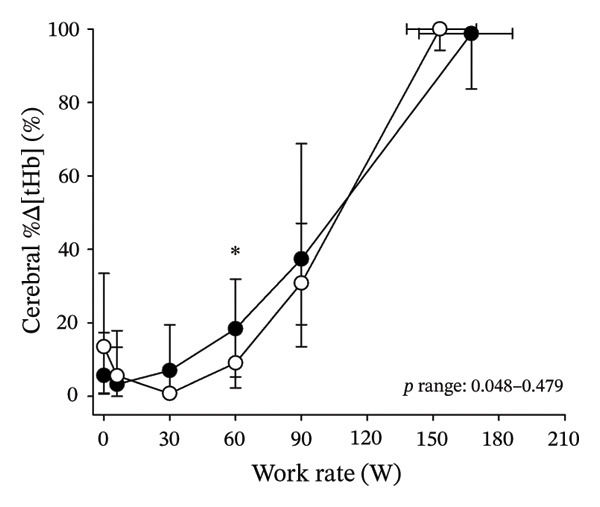
(h)

Cerebral oxygenation parameters (TSI, Δ[HHb], Δ[tHb]) at the relative work rate of 50% did not have univariate associations with HOMA‐IR (*p* = 0.684, *p* = 0.484, *p* = 0.406, respectively).

Table 3 presents the results of the linear regression analyses used to identify potential predictors of RPE/MET slope in the whole pooled population. TSI, Δ[HHb] or Δ[tHb] at a relative work rate of 50% did not have univariate associations with RPE/MET slope. On the contrary, BMI, HOMA‐IR and FEV1 (z‐score) had univariate associations with RPE/MET slope, but in the multivariate regression analysis, only high BMI and low FEV1 (z‐score) predicted high RPE/MET slope independently.

**TABLE 3:** Factors associated with RPE/MET slope^a^ (*n* = 48).

	**Univariate analysis**		Multivariate analysis**^b^**	
	**Standardised B**	**p**	**Standardised B**	**p**

Age (years)	−0.204	0.164	—	—
LTPA (h:min·wk^−1^)	−0.258	0.083	—	—
BMI (kg·m^−2^)	0.563	< 0.001	0.537	< 0.001
FEV1 (z‐score)	−0.464	0.001	−0.432	< 0.001
HOMA‐IR	0.411	0.004	—	—
TSI at 50% (%)	−0.141	0.339	—	—
Δ [HHb] at 50% (µmol·L^−1^)	−0.065	0.658	—	—
Δ [tHb] at 50% (µmol·L^−1^)	−0.161	0.273	—	—

Abbreviations: Δ[HHb] at 50%, relative concentration change of cerebral deoxygenated haemoglobin at the relative work rate of 50%; Δ[tHb] at 50%, relative concentration change of cerebral total haemoglobin at the relative work rate of 50%; BMI, body mass index; FEV1, forced expired volume in 1 s; HOMA‐IR, homeostasis model assessment index of insulin resistance; LTPA, leisure‐time physical activity; TSI, cerebral tissue saturation index at the relative work rate of 50%.

^a^ = Ln transformed for statistical analysis due to non‐normally distributed data.

^b^ = Variables having a univariate association with RPE/MET slope at *p* < 0.05 were selected in the multivariate backward stepwise linear regression analysis. The variables presented here include only those that showed an independent association (*p* < 0.05) with RPE/MET slope.

Furthermore, the results of the sensitivity analyses demonstrate that there were no differences in cerebral (de)oxygenation responses as a function of absolute or relative work rates between the subjects belonging to the low and high extremities of HOMA‐IR or RPE/MET slope spectra (Supporting Information 3).

## 4. Discussion

The purpose of this study was to examine the associations of IR to perceived exertion and cerebral oxygenation during acute incremental dynamic exercise. The novel findings of this study were threefold: (1) IR was not associated with perceived exertion during incremental cycling, (2) IR was not associated with cerebral oxygenation during incremental cycling, and (3) perceived exertion was not associated with cerebral oxygenation during incremental cycling. These findings are based on both group comparisons and whole‐population linear regression analyses and together suggest that individuals with IR do not experience pronounced perceived exertion and are not exposed to jeopardised cerebral oxygenation during cycling compared to their counterparts with normal glucose–insulin homeostasis.

### 4.1. IR and Perceived Exertion

We examined associations between IR and perceived exertion during incremental cycling in various ways. First, we divided subjects into two different groups based on HOMA‐IR (i.e., HOMA_LOW_ and HOMA_HIGH_), compared the groups’ RPE responses to incremental cycling at both absolute and relative work rates, and did not observe any between‐group differences. Meanwhile, the RPE/MET slope was steeper in HOMA_HIGH_ than in HOMA_LOW_ and it is noteworthy that 10 of the 14 subjects of the HOMA_HIGH_ group belonged to the RPE/MET_HIGH_ group. Second, in addition to such a dichotomous group‐comparison approach, we used univariate and multivariate linear regression analyses in the whole pooled population to identify independent predictors of pronounced perceived exertion reflected by the RPE/MET slope outcome. In line with the dichotomous group comparison, the regression analyses showed a positive univariate association between HOMA‐IR and RPE/MET slope. However, most importantly, the multivariate regression analysis revealed that not HOMA‐IR but only high BMI and low FEV1 (z‐score) predicted high RPE/MET slope independently. Taken together, these findings do not support an independent association between IR and perceived exertion during exercise and are largely parallel to previous studies performed in individuals with T2D: With the exception of one single study by Huebschmann et al. [10], who reported higher RPE during submaximal cycling in women with T2D compared to women with no diabetes, other previous studies do not support that there are significant differences in RPE responses during exercise between individuals with T2D and their matched controls [11, 12].

Instead of any independent association between IR and perceived exertion during exercise, our findings suggest the nonindependent association was mediated by the confounding effect of body size. Indeed, our findings imply pronounced perceived exertion in this cohort was particularly linked with excess body size, which may exert limitations to breathing capacity in many ways (e.g., decreased lung function, altered respiratory mechanics, increased work of breathing, pronounced metabolic demands of exercise) [29]. In addition, we observed pronounced perceived exertion to be independently linked also with lower FEV1, which is plausible as FEV1 reflects airway resistance, elastic recoil of the lung and respiratory muscle strength [30], but it is also related to a multitude of clinical, demographic and social factors, including cognitive characteristics [31].

### 4.2. IR and Cerebral Oxygenation

Cerebral NIRS responses to incremental exercise have been described earlier and overall reflect cerebral deoxygenation along with increasing exercise intensity: As a sign of developing imbalance between local cerebral O_2_ delivery and utilisation, [HHb] increases towards hard and very hard intensities so that the timing of acceleration of the increase depends on individual’s fitness level, while TSI shows a decreasing trend towards hard and very hard intensities [32]. As cerebral oxygenation is tightly coupled with cerebral blood flow and its regulators during exercise [16], and as IR has been proposed to impair particularly cerebrovascular reactivity [17], endothelial function and insulin‐mediated vasodilation also during exercise [20], we were interested in examining potential associations between IR and cerebral (de)oxygenation during exercise.

In general, the observed patterns of cerebral (de)oxygenation responses in this study conformed to those reported in existing literature [32]. In terms of IR, we examined associations between IR and cerebral oxygenation in two ways: on the one hand, with group comparisons (i.e., HOMA_LOW_ vs. HOMA_HIGH_), and on the other hand, with univariate linear regression analyses among the whole pooled population. Additionally, we also performed sensitivity analyses to compare cerebral oxygenation during exercise between the subjects belonging to the low and high extremities of the HOMA‐IR groups. None of these statistical approaches supported associations between IR and cerebral oxygenation during exercise. This is consistent with the fact that we did not observe any differences in key regulators of cerebral blood flow (i.e., estimated PaCO_2_, CaO_2_, MAP and CO) between HOMA_LOW_ and HOMA_HIGH_ groups during cycling. On the other hand, if any between‐group differences existed in the rest of the regulators of cerebral blood flow (i.e., cerebral CO_2_‐induced vasodilation, cerebral metabolism and neurogenic activity), which were not measured here, such differences were not large enough to lead to observable between‐group differences in dynamic cerebral oxygenation.

### 4.3. Perceived Exertion and Cerebral Oxygenation

As our third study question, we examined associations between perceived exertion and cerebral (de)oxygenation during incremental cycling in various ways. As a key variable in this regard, we used RPE/MET slopes, which we drew for each individual. To the best of our knowledge, determining such slopes is a novel way to approach perceived exertion and can be regarded as justified, as it normalises the increasing exertion perception against the increase in body mass‐adjusted metabolic cost. First, we divided subjects into two groups (i.e., RPE/MET_LOW_ and RPE/MET_HIGH_) based on the median value of individual RPE/MET slopes, compared the groups’ cerebral (de)oxygenation responses to incremental cycling at both absolute and relative work rates, and did not observe any between‐group differences. Second, in addition to such a dichotomous approach, we performed univariate linear regression analyses and observed the RPE/MET slope did not have any associations with cerebral NIRS parameters at a relative work rate of 50%. Third, we also performed sensitivity analyses to compare cerebral oxygenation during exercise between the subjects belonging to the low and high extremities of RPE/MET slope spectra and did not observe differences in cerebral NIRS responses between those with low and high RPE/MET slopes.

Impaired cerebral oxygenation during exercise might cause a reduction in efferent motor drive, thereby impairing skeletal muscle activation and thus increasing perceived exertion [14]. Our data do not disagree with such a hypothesis. Instead, it is noteworthy that as the subjects in this study were free of chronic diseases or other clinically relevant conditions (instead of overweight or obesity in overweight or obese individuals), neither IR nor any other biological characteristic of the subjects impaired cerebral oxygenation responses to a pathophysiological extent. Thus, it is not surprising that we did not observe any associations between perceived exertion and cerebral NIRS parameters. Instead, we observed pronounced perceived exertion to be independently associated with high BMI and low FEV1, as discussed previously.

### 4.4. Methodological Considerations

This study has its strengths and limitations. 144 subjects were originally assessed for eligibility, and a total of 96 subjects were excluded, as they did not meet our inclusion criteria. A relatively strict exclusion process was essential, as the key focus of our study questions was on the associations between glucose–insulin homeostasis and perceived exertion and cerebral oxygenation during exercise, and conditions such as cardiovascular or respiratory diseases would have distorted the outcome. Furthermore, our retrospectively determined cohort proved to be heterogeneous in terms of HOMA‐IR (range: 0.6–6.6), which is also important, as the relatively wide range of HOMA‐IR enables responding to such a focus. On the other hand, due to the exclusion process, these results are considerable only among premenopausal women without clinically overt cardiovascular or respiratory diseases. It is also important that we were able to draw a comprehensive picture of the subjects’ respiratory and cardiovascular adjustments to acute exercise as we combined breath‐by‐breath ventilatory gas exchange measurements with simultaneous pulse oximetry, impedance cardiography and blood pressure measurements. Furthermore, our statistical analyses combined group comparisons with whole‐population uni‐ and multivariate linear regression analyses, and as the results of both of these statistical approaches support each other, it is safe to draw conclusions from our data; for example, when there is no linear association between an outcome of interest and some other variable in a pooled whole population, Type II error is unlikely in terms of such group comparisons, which show a lack of between‐group difference in the outcome of interest.

Using HOMA‐IR to evaluate the level of IR may be regarded as the main limitation of this study. This is because HOMA‐IR is based on fasting levels of plasma glucose and serum insulin and thus does not allow evaluation of IR based on glucose‐stimulated response [33]. Consequently, HOMA‐IR does not distinguish IR from primary hyperinsulinaemia but is rather a single measure of unexplained fasting hyperinsulinaemia [34]. However, while we do recognise such limitations related to underlying glucose–insulin (patho)physiology, a comprehensive meta‐analysis has shown that the widely used HOMA‐IR correlates well with whole‐body glucose disposal rate (M‐value) based on the gold standard hyperinsulinaemic–euglycaemic clamp (*r = *−0.60, 95% CI ‐0.66–0.53), making it one of the best indirect alternatives to evaluate IR when the clamp method is not available [35]. There are also considerations related to using NIRS: First, as it interrogates structures located 2‐3 cm below the surface of the skull, NIRS does not enable examining all or deeper brain regions involved in emotional and cognitive functions. Second, we set the NIRS optode at the level of the LPFC, which is far from covering all cortical areas. However, importantly, LPFC has been linked with planning [24] and pacing [25] of voluntary movements and may also contribute to exercise (in)tolerance and termination [26]. Furthermore, the cross‐sectional design is also a limitation of this study as it does not enable drawing inferences related to the causality of the associations of interest.

### 4.5. Conclusions

These results suggest that IR is not independently associated with perceived exertion or cerebral oxygenation responses during cycling. In addition, we did not observe associations between perceived exertion and cerebral oxygenation during exercise in this population consisting of premenopausal women without clinically overt respiratory or cardiovascular diseases. In terms of perceived exertion during exercise, we found higher BMI and lower FEV1 (z‐score) to be independently associated with higher subjectively perceived exertion, highlighting the particularly significant effects of body size and lung function on exercise‐related psychological sensations.

## Author Contributions

Juuso Rissanen, Antti‐Pekka E. Rissanen and Juha E. Peltonen made important contributions to the conception and design of the study. Juuso Rissanen, Antti‐Pekka E. Rissanen and Juha E. Peltonen made important contributions to the acquisition and analysis of data, and all authors made important contributions to the interpretation of data. Juuso Rissanen and Antti‐Pekka E. Rissanen drafted the manuscript, and all other authors revised the manuscript critically for intellectual content.

## Funding

The funding for this study was provided by Tekes–the Finnish Funding Agency for Technology and Innovation (40043/07), Business Finland (575/31/2015), the Ministry of Education and Culture (Finland), University of Helsinki, Diamond (TYH2016215), Helsinki Deaconess Institute–Diacor Terveyspalvelut Oy, Diaconia University of Applied Sciences, Amer Sports Oy, HUR Oy and Firstbeat Technologies Oy. Open access publishing facilitated by Ita‐Suomen yliopisto, as part of the Wiley ‐ FinELib agreement.

## Disclosure

All authors approved the final version of the manuscript.

## Conflicts of Interest

The authors declare no conflicts of interest.

## Supporting Information

Additional supporting information can be found online in the Supporting Information section.

## Supporting information


**Supporting Information** Supporting information 1: Flowchart illustrating the inclusion process. Supporting information 2: Detailed description of applied CPET methodology. Supporting information 3: Figures illustrating cerebral (de)oxygenation responses during exercise in subjects belonging to the low and high extremities of HOMA‐IR (Figure 1) or RPE/MET slope (Figure 2) spectra.

## Data Availability

The data that support the findings of this study are available from the corresponding author upon reasonable request.
