# Supplementary material for: Insulin Resistance Is Not Associated With Perceived Exertion or Cerebral Oxygenation During Exercise in Women
Source: Transl Sports Med. 2026 May 31;2026:7516755. doi: 10.1155/tsm2/7516755 (PMC13240380; doi:10.1155/tsm2/7516755)
Supplement: Supplementary file 1 — Supporting Information Supporting information 1: Flowchart illustrating the inclusion process. Supporting information 2: Detailed description of applied CPET methodology. Supporting information 3: Figures illustrating cerebral (de)oxygenation responses during exercise in subjects belonging to the low and high extremities of HOMA‐IR (Figure 1) or RPE/MET slope (Figure 2) spectra. [file TSM2-2026-7516755-s001.docx]

**Supplementary Material 1: Flow chart illustrating the inclusion process**


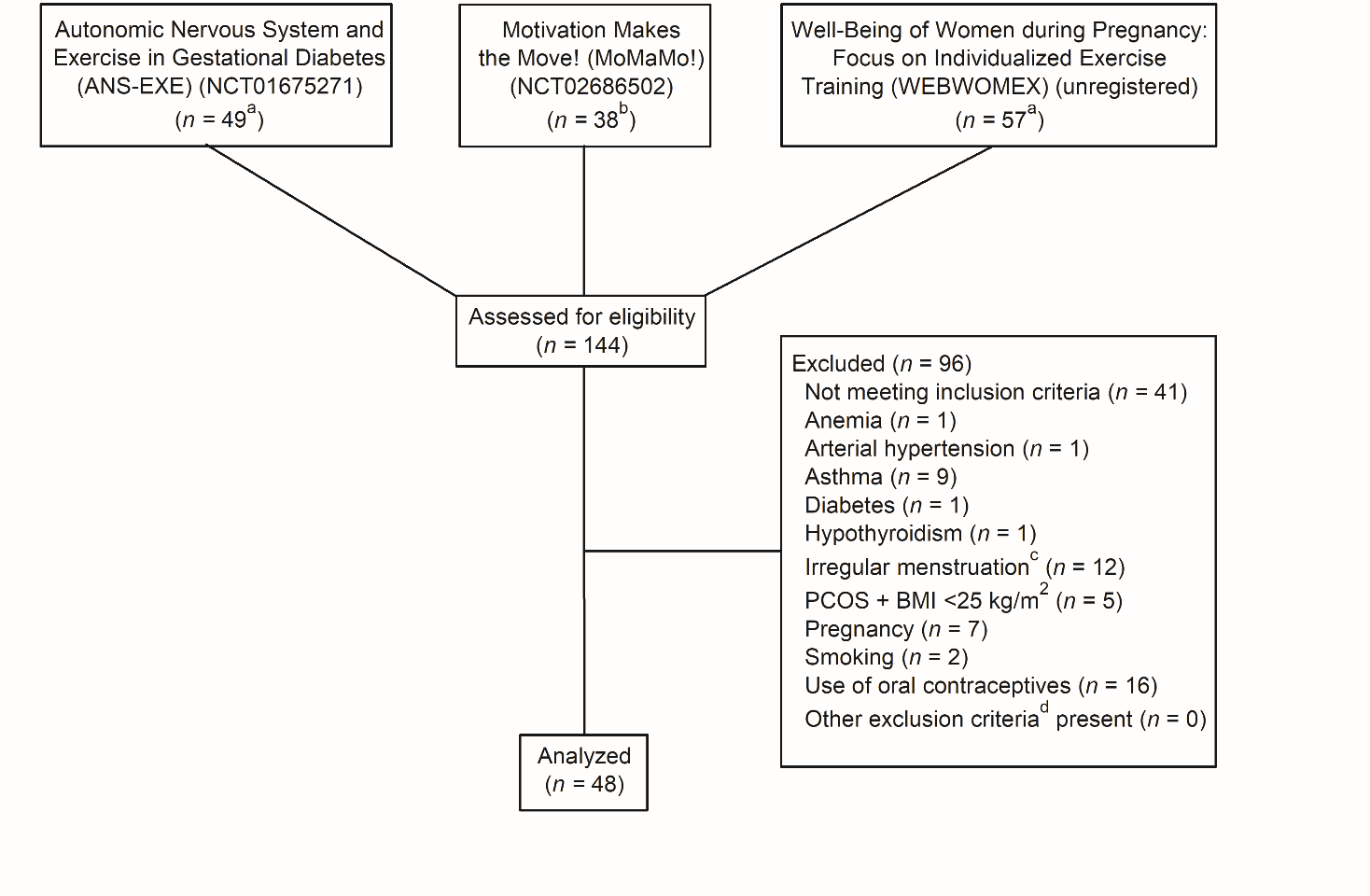


**FLOW CHART** Flow diagram of the participant inclusion and exclusion. ^a^ Includes the participants with performed cardiopulmonary exercise test at the baseline of ANS-EXE or WEBWOMEX; ^b^ Includes the participants with performed cardiopulmonary exercise test at the baseline of MoMaMo! by the estimated MoMaMo! completion date (i.e., May 2019); ^c^ Menstrual period not occurring every 23-32 days (Cole et al., 2009); ^d^ Androgen-secreting tumors, antiandrogen medication, betablocker medication, congenital adrenal hyperplasia, Cushing’s syndrome, medication influencing glucose homeostasis, physical disability, substance abuse, and/or any cardiovascular, endocrine, musculoskeletal, neurological, or respiratory disease that could have affected O_2_ uptake, convection, and/or diffusion. BMI, body mass index; PCOS, polycystic ovary syndrome.

**Supplementary Material 2: Detailed description of applied CPET methodology**

Breath-by-breath ventilation was measured by a low-resistance turbine (Triple V, Jaeger Mijnhardt, Bunnik, The Netherlands) to determine inspiratory and expiratory gas volumes and flow during the test. The turbine was calibrated before each test by using a syringe of 3.00 L volume (Hans Rudolph, Inc., Kansas City, MO). In ANS-EXE and WEBWOMEX, inspired and expired gases were sampled continuously at mouth and analyzed for concentrations of O_2_, CO_2_, N_2_ and Ar by mass spectrometry (AMIS 2000, Innovision A/S, Odense, Denmark) after calibration with precision-analyzed gas mixtures. Breath-by-breath respiratory data were collected as raw data. The raw data were transferred to a computer, which determined gas delays for each breath to align concentrations with volume data and to build a profile of each breath. Breath-by-breath alveolar gas exchange was then calculated with the AMIS algorithms, and the data were interpolated to obtain second-by-second values. In MoMaMo!, breath-by-breath pulmonary ventilation and alveolar gas exchange were obtained by combining the low-resistance turbine with Oxycon Pro metabolic cart (VIASYS Healthcare GmbH, Hoechberg, Germany).

Minute ventilation/pulmonary CO_2_ output (V̇E/V̇CO_2_) slope was determined by performing a linear regression of breath-by-breath V̇E over breath-by-breath V̇CO_2_ from the exercise onset to ventilatory threshold 2 (VT2) (or to peak exercise if VT2 was undetectable) (Neder et al., 2017); VT2 was determined identifying the starting point of a consistent rise in V̇E/V̇CO_2_ and a consistent fall in end-tidal pressure of CO_2_ of respired gases (PETCO_2_). Non-invasively estimated PaCO_2_ was determined with the following equation (Jones et al., 1979): Estimated PaCO_2_ = 5.5 + 0.9 × PETCO_2_ - 2.1 × tidal volume. Respiratory exchange ratio (RER) was calculated as a quotient of V̇CO_2_ and V̇O_2_ (Edvardsen et al., 2014). Rating of perceived exertion (RPE) was obtained using the Borg category scale (6–20) (Borg, 1970) at the end of each work rate and at peak exercise.

Heart rate (HR) and the electrical activity of the heart were monitored by ECG (PowerLab, ADInstruments, Oxford, United Kingdom), and left ventricular stroke volume (SV) was evaluated by a non-invasive impedance cardiograph device (PhysioFlow, Manatec Biomedical, Paris, France) throughout CPET. The estimate of cardiac output (CO) was calculated by multiplying SV with HR. This method has been shown to provide valid and reliable data on SV and CO at rest and during low-to-maximal intensity exercise in individuals with normal weight, overweight, and obesity (Charloux et al., 2000; Richard et al., 2001). In addition, the method has been acknowledged by the current clinical CPET recommendation of the European Association for Cardiovascular Prevention and Rehabilitation and the American Heart Association (Guazzi et al., 2016). Systolic and diastolic blood pressures were measured automatically (Tango+, SunTech Medical, Morrisville, USA) from the brachial artery at seated rest and at the end of each work rate. Mean arterial pressure (MAP) was calculated: MAP = (systolic arterial pressure + 2 × diastolic arterial pressure) / 3. Systemic vascular conductance (SVC) was calculated as the inverse of systemic vascular resistance, which was calculated according to Darcy’s law: SVC = 1 / ((MAP - central venous pressure) / CO), where central venous pressure was assumed to be 0 mmHg (Mortensen et al., 2005). Fingertip pulse oximetry (Nonin 9600, Nonin Medical, Inc., Plymouth, USA) was used to monitor arterial O_2_ saturation (SpO_2_). Arterial O_2_ content (CaO_2_) was calculated as the product of SpO_2_, [Hb], and the physiological O_2_ binding coefficient of Hb (1.34 ml g^-1^): CaO_2_ = SpO_2_ × [Hb] × 1.34. Systemic arteriovenous O_2_ difference (C(a-v)O_2_) was derived using the Fick equation: C(a-v)O_2_ = V̇O_2_/CO. Noteworthily, determination of SV, CO, or SpO_2_ during exercise, and thereby determination of any other variable calculated based on the three variables, depends significantly on the applied method. In this regard, while the PhysioFlow method applied here is a feasible method to determine SV and CO during exercise compared to many others (Siebenmann et al., 2015) as does widely used fingertip pulse oximetry, C(a-v)O_2_ was physiologically implausibly observed to be higher than CaO_2_ at a single cycling work rate in nine subjects; this is a result of single inaccurate values of SV, CO, and/or SpO_2_, and the data on the nine subjects were not included in the analyses of SV, CO, SVC, and C(a-v)O_2_, and are not reported here.

To avoid any erroneous interpretations caused by body size and composition between HOMA-IR or RPE/MET groups, we sought optimal variable-specific exponents of denominators to which to scale V̇O_2_, SV, CO, and SVC. We set three prerequisites for such denominators (Turley et al., 2006): 1) An absolute value of a scaled variable (e.g., V̇O_2_ in l min^-1^) must be linearly associated with its scaling denominator (e.g., fat-free mass (FFM)), 2) a scaled value (e.g., ml min^-1^ kg^-1^ FFM) must not be linearly associated with its scaling denominator (e.g., FFM), and 3) a regression line for an absolute value of a scaled variable (e.g., V̇O_2_ in l min^-1^) versus its scaling denominator (e.g., FFM) must travel through the origin (i.e., 95% CI of the y intercept of the regression line must include 0). For V̇O_2peak_, V̇O_2peak_ scaled to FFM (i.e., ml min^-1^ kg^-1^ FFM) met these three prerequisites, which was in accordance with extensive literature (Lolli et al., 2017). Instead, peak SV and peak CO scaled to body surface area or FFM did not meet the second or the third prerequisites, which made us perform allometric scaling based on the log-linear regression model as described by Welsman et al., (1996): The allometric equations took the form of ln y = ln a + b · ln x, in which y represents a dependent variable (i.e., SV or CO), a represents a constant, b represents unstandardised β (which will eventually be determined and represent the optimal exponent of a scaling denominator variable), and x presents an independent variable (i.e., FFM). This way, we found that the optimal scaling variables for peak SV and peak CO were FFM^-0.55^ and FFM^-0.50^, respectively. In other words, peak SV presented as ml kg^-0.55^ FFM and peak CO presented as ml min-1 kg^-0.50^ FFM met all the above-mentioned three prerequisites and were thus independent of body size and composition. SVC turned out be independent of body size and composition and needed no scaling.

**Supplementary Material 3: Figures illustrating cerebral (de)oxygenation responses during exercise in subjects belonging to the low and high extremities of HOMA-IR (Figure 1) or RPE/MET slope (Figure 2) spectra**

**
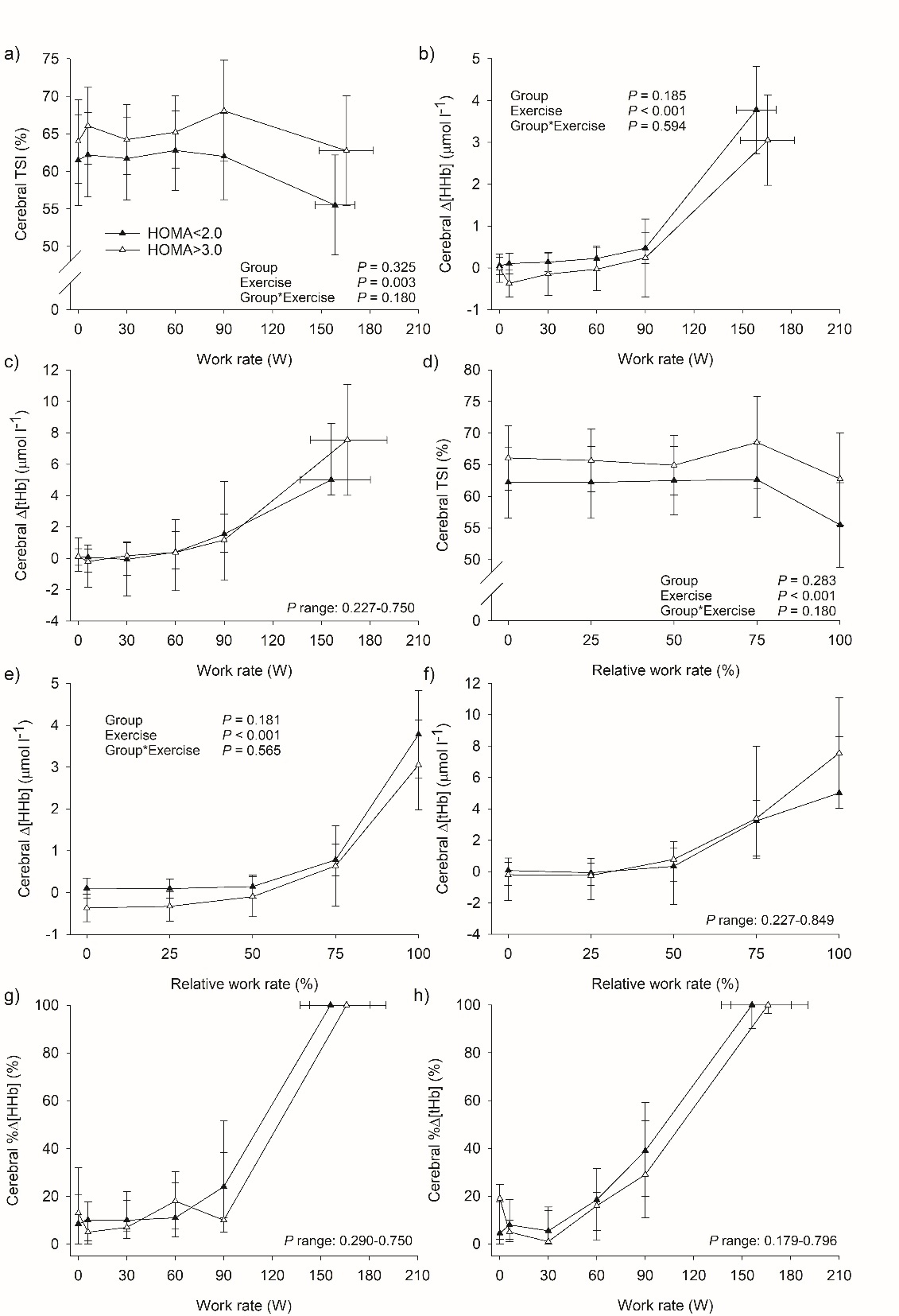
**

**SUPPLEMENTARY MATERIAL 3 - FIGURE 1** Cerebral tissue saturation index (TSI) as a function of work rate (W) (a), relative concentration change of cerebral deoxygenated Hb (Δ[HHb]) as a function of work rate (W) (b), relative concentration change of cerebral total Hb (Δ[tHb]) as a function of work rate (W) (c), cerebral TSI as a function of relative work rate (%) (d), cerebral Δ[HHb] as a function of relative work rate (%) (e), cerebral Δ[tHb] as a function of relative work rate (%) (f), normalised relative concentration change of cerebral deoxygenated Hb (%Δ[HHb]) as a function of work rate (W) (g), normalised relative concentration change of cerebral total Hb (%Δ[tHb]) as a function of work rate (W) (h). Black triangles (▲) = HOMA<2.0 (n = 24), white triangles (∆) = HOMA>3.0 (n = 13). The *P* values refer to a two-way repeated-measures ANOVA: Group (HOMA<2.0 vs. HOMA>3.0) is a between-subjects factor and Exercise (panel a: rest, unloaded cycling, work rates accomplished by each subject [30 W, 60 W, 90 W] and peak exercise; panel d: rest and 25-100 % of peak work rate) is a within-subject factor. *P* range describes the range of the *P* values from minimum to maximum in Mann-Whitney U test.

**
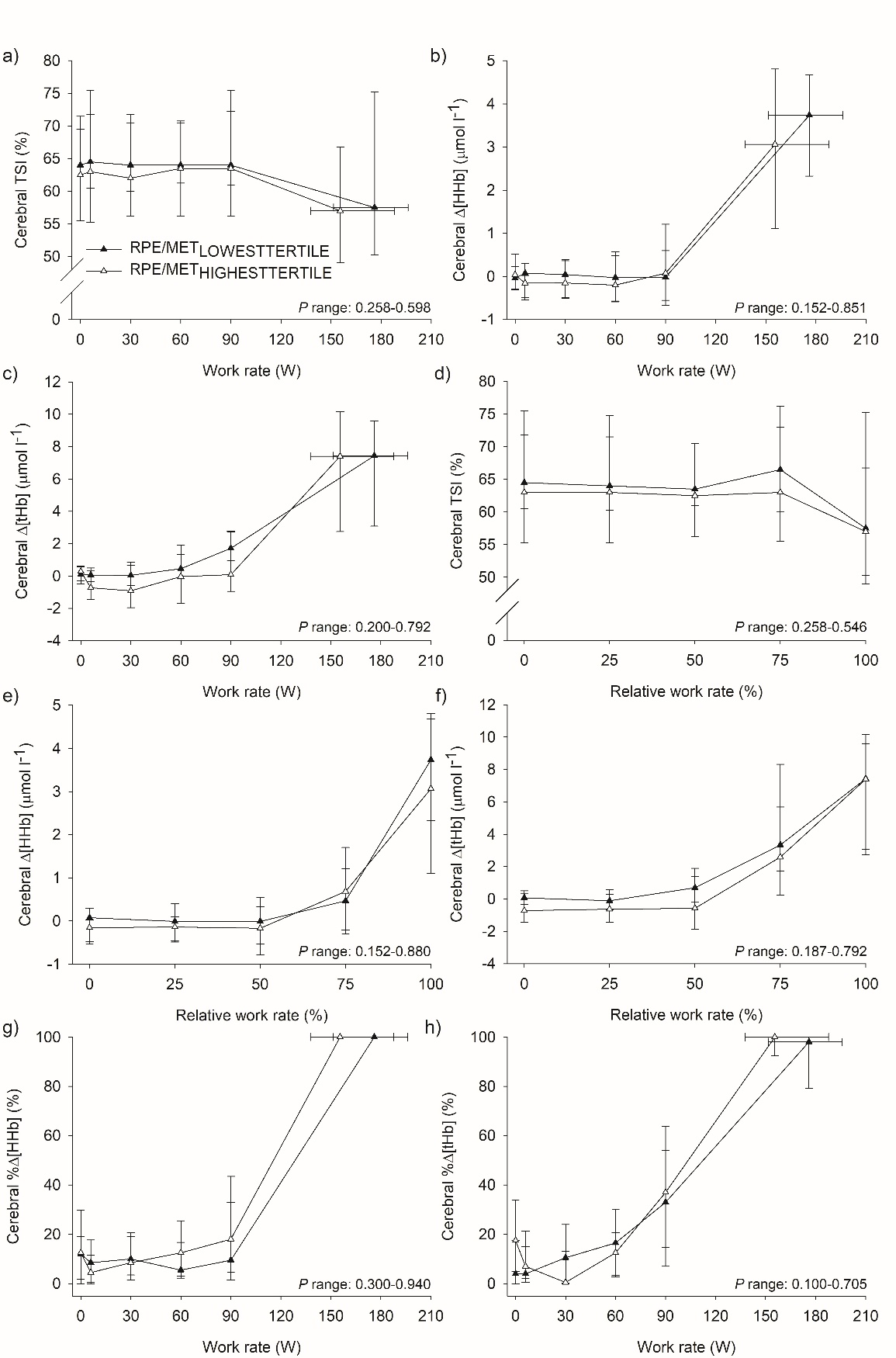
**

**SUPPLEMENTARY MATERIAL 3 - FIGURE 2** Cerebral tissue saturation index (TSI) as a function of work rate (W) (a), relative concentration change of cerebral deoxygenated Hb (Δ[HHb]) as a function of work rate (W) (b), relative concentration change of cerebral total Hb (Δ[tHb]) as a function of work rate (W) (c), cerebral TSI as a function of relative work rate (%) (d), cerebral Δ[HHb] as a function of relative work rate (%) (e), cerebral Δ[tHb] as a function of relative work rate (%) (f), normalised relative concentration change of cerebral deoxygenated Hb (%Δ[HHb]) as a function of work rate (W) (g), normalised relative concentration change of cerebral total Hb (%Δ[tHb]) as a function of work rate (W) (h). Black triangles (▲) = RPE/MET_LOWESTTERTILE_ (n = 16), white triangles (∆) = RPE/MET_HIGHESTTERTILE_ (n = 16). *P* range describes the range of the *P* values from minimum to maximum in Mann-Whitney U test.
